# Supplementary figures and images for: Stenoparib, an Inhibitor of Cellular Poly(ADP-Ribose) Polymerase, Blocks Replication of the SARS-CoV-2 and HCoV-NL63 Human Coronaviruses In Vitro
Source: mBio. 2021 Jan 19;12(1):e03495-20. doi: 10.1128/mBio.03495-20 (PMC7845641; doi:10.1128/mBio.03495-20)

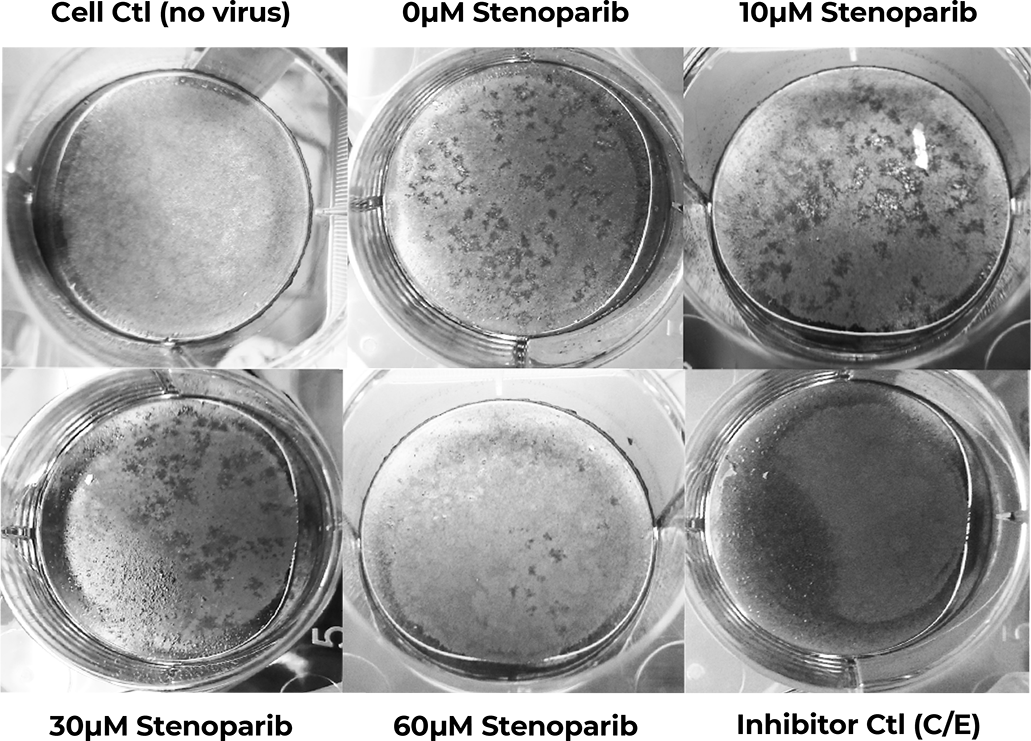

Supplement: FIG S1 [file mBio.03495-20-sf001.tif]
